# Identification of a 3^rd^ Na^+^ Binding Site of the Glycine Transporter, GlyT2

**DOI:** 10.1371/journal.pone.0157583

**Published:** 2016-06-23

**Authors:** Nandhitha Subramanian, Amanda J. Scopelitti, Jane E. Carland, Renae M. Ryan, Megan L. O’Mara, Robert J. Vandenberg

**Affiliations:** 1 Research School of Chemistry, The Australian National University, Canberra, ACT, 2601, Australia; 2 Discipline of Pharmacology, School of Medical Sciences, University of Sydney, Sydney, NSW, 2006, Australia; 3 Department of Physiology and Biophysics, Weill Cornell Medical College, New York, NY, 10021, United States of America; University of Cambridge, UNITED KINGDOM

## Abstract

The Na^+^/Cl^-^ dependent glycine transporters GlyT1 and GlyT2 regulate synaptic glycine concentrations. Glycine transport by GlyT2 is coupled to the co-transport of three Na^+^ ions, whereas transport by GlyT1 is coupled to the co-transport of only two Na^+^ ions. These differences in ion-flux coupling determine their respective concentrating capacities and have a direct bearing on their functional roles in synaptic transmission. The crystal structures of the closely related bacterial Na^+^-dependent leucine transporter, LeuT_Aa_, and the *Drosophila* dopamine transporter, dDAT, have allowed prediction of two Na^+^ binding sites in GlyT2, but the physical location of the third Na^+^ site in GlyT2 is unknown. A bacterial betaine transporter, BetP, has also been crystallized and shows structural similarity to LeuT_Aa_. Although betaine transport by BetP is coupled to the co-transport of two Na^+^ ions, the first Na^+^ site is not conserved between BetP and LeuT_Aa_, the so called Na1' site. We hypothesized that the third Na^+^ binding site (Na3 site) of GlyT2 corresponds to the BetP Na1' binding site. To identify the Na3 binding site of GlyT2, we performed molecular dynamics (MD) simulations. Surprisingly, a Na^+^ placed at the location consistent with the Na1' site of BetP spontaneously dissociated from its initial location and bound instead to a novel Na3 site. Using a combination of MD simulations of a comparative model of GlyT2 together with an analysis of the functional properties of wild type and mutant GlyTs we have identified an electrostatically favorable novel third Na^+^ binding site in GlyT2 formed by Trp263 and Met276 in TM3, Ala481 in TM6 and Glu648 in TM10.

## Introduction

The two glycine transporters, GlyT1 and GlyT2, differ in their concentrating capacity [1]; play distinct roles in regulating neurotransmission [2]; and are also the targets for novel pharmaceuticals for the treatment of schizophrenia [3] and chronic pain [4]. GlyT1 and GlyT2 belong to the solute carrier family SLC6 that also includes transporters for the neurotransmitters GABA, serotonin, norepinephrine and dopamine [5, 6]. Members of this family of transporters couple the transport of neurotransmitter to the co-transport of Na^+^ and Cl^-^ ions, with the transport of glycine by GlyT1 being coupled to two Na^+^ ions, while glycine transport by GlyT2 is coupled to three Na^+^ ions. These differences in ion-substrate flux coupling allow glycine transporters to serve different roles in the regulation of glycine concentrations in the central nervous system [1]. At excitatory synapses, GlyT1 maintains extracellular synaptic glycine concentrations at ~100 nM and small fluctuations in ion gradients across the cell membrane can reduce the concentrating capacity of GlyT1, elevating synaptic glycine [7, 8]. Conversely, GlyT2 operates to concentrate glycine in presynaptic neurones, which is necessary for glycine storage in synaptic vesicles for neurotransmission [8, 9].

Our current understanding of the structure and function of SLC6 transporters has been greatly enhanced by the determination of the crystal structures of the sodium-dependent bacterial leucine transporter, LeuT_Aa_ [10], and the *Drosophila* dopamine transporter, dDAT [11]. Two homologous Na^+^ binding sites have been identified in both LeuT_Aa_ [10] and dDAT [11]. For both LeuT_Aa_ and dDAT, the first (Na1) is found between unwound regions of transmembrane domains 1 (TM1) and 6 (TM6), while the second (Na2) is formed by TM1 and TM8. Molecular dynamics simulations and mutagenesis studies have demonstrated that both Na1 and Na2 of LeuT_Aa_ are conserved in GlyT2 [12]. Similarly, these sites are conserved in the GABA, dopamine (dDAT) and serotonin transporters [6]. The third Na^+^ binding site (Na3) on GlyT2 remains to be identified. The recent crystal structure of the sodium dependent betaine transporter, BetP, shows that despite its low level of sequence identity with other SLC6 transporter structures, it shares the conserved LeuT_Aa_ fold characteristic of these transporters [13]. Furthermore, substrate transport by BetP is also coupled to the co-transport of two Na^+^ ions. Intriguingly, whilst BetP contains a Na^+^ binding site corresponding to Na2 of LeuT_Aa_, it does not possess the Na1 site. Instead, BetP contains a unique Na^+^ binding site, termed Na1' [14, 15]. We hypothesize that the Na3 of GlyT2 corresponds to the Na1' site of BetP, in close proximity to S280 and A284 (TM3) of GlyT2. Here we use molecular dynamics (MD) simulations of a membrane-embedded, refined GlyT2 homology model and comparative analysis of the functional properties of wild type and mutant glycine transporters to identify a potential third Na^+^ binding site on GlyT2.

## Materials and Methods

### Comparative modeling

Due to the relatively low sequence identity between the human glycine transporters and other members of the SLC6 superfamily for which structural data is available, a homology model of the GlyT2 glycine transporter based on the outward-occluded dDAT structure (PDBid: 4M48) was developed using a protein fold recognition (or threading) approach, as implemented in the Phyre2 webserver [16]. In this approach, the amino acid sequence of GlyT2 was compared to a non-redundant database of protein structures from the Structural Classification of Proteins (SCOP) database and the Protein Data Bank (PDB) to identify homologues. Further secondary structure predictions on the GlyT2 sequence are used to increase the accuracy of the sequence alignment with homologues from the structural database prior to model building using Phyre2 webserver [16]. GlyT2 shares 50% sequence identity with dDAT. The amino acid sequence alignment of Yamashita et al. [17] was used in verification of the resulting GlyT2 homology model prior to its use in MD simulations. The multiple sequence alignments generated during the development of the GlyT2 homology model are provided as Supporting Information (Fig A in S1 File and S2, S3 and S4 Files).

The N-terminal 189 residues, the C-terminal 53 residues and residues from W315 to Q362 in EL2 of GlyT2 were removed from the homology model because these regions cannot be modeled on dDAT. The ends of the protein model have been capped by protonating the C-termini and deprotonating the N-termini to avoid the introduction of inappropriate charges within the protein. The pKa values of all ionizable groups were assessed using the PROPKA server [18, 19] and the initial protonation states of the relevant groups at neutral pH were assigned accordingly. PROPKA identifies all ionizable groups in the structure and calculates the distance between the ionizable residue and all neighboring residues that may be involved in hydrogen bonding. Initial pK_a_’s were assigned to ionizable groups and these values were iteratively refined to give a self-consistent pKa based on hydrogen bonding patterns and the accessibility of the residue to solvent [18, 19]. On the basis of the pKa values predicted using the PROPKA server [18, 19] all histidine residues were predicted to be predominately singly protonated at pH 7.0. Aspartate, glutamate, arginine and lysine residues were charged.

### Molecular dynamics simulation

The GROMACS [20] version 3.3.3 molecular dynamics package in conjunction with the GROMOS 54A7 force field [21] was used in all MD simulations. Water was represented explicitly using the simple point charge (SPC) model [22]. Each system was simulated under periodic boundary conditions in a rectangular simulation box. The temperature of the system was maintained by coupling the protein and lipids together and the solvent, ions and the ligand together to an external temperature bath at 300 K with a coupling constant of τ_T_ = 0.1 ps using a Berendsen thermostat [23]. The pressure was maintained at 1 bar by weakly coupling the system to a semi-isotropic pressure bath using an isothermal compressibility of 4.5x10^-5^ bar^-1^ and a coupling constant of τ_P_ = 0.5 ps. During the simulations, the length of all bonds within the protein and lipids were constrained using the LINCS algorithm [24]. The SETTLE algorithm [25] was used to constrain the geometry of water molecules. In order to further extend the timescale that could be simulated, the mass of hydrogen atoms was increased to 4 a.m.u. by transferring mass from the atom to which it was attached. This allows a time step of 4 fs to be used to integrate the equation of motion without significantly affecting the thermodynamic properties of the system [26]. Non-bonded interactions were calculated using a twin-range cut-off. Interactions within the short-range cut-off of 0.8 nm were updated every time step. Interactions within the longer-range cut-off of 1.4 nm were updated every 5 time steps, together with the pair list. To correct for the truncation of electrostatic interactions beyond the 1.4 nm long-range cut-off, a reaction field correction was applied using an effective dielectric permittivity value (ε_r_) of 78.5 [27].

#### System set-up

**Fig 1:**
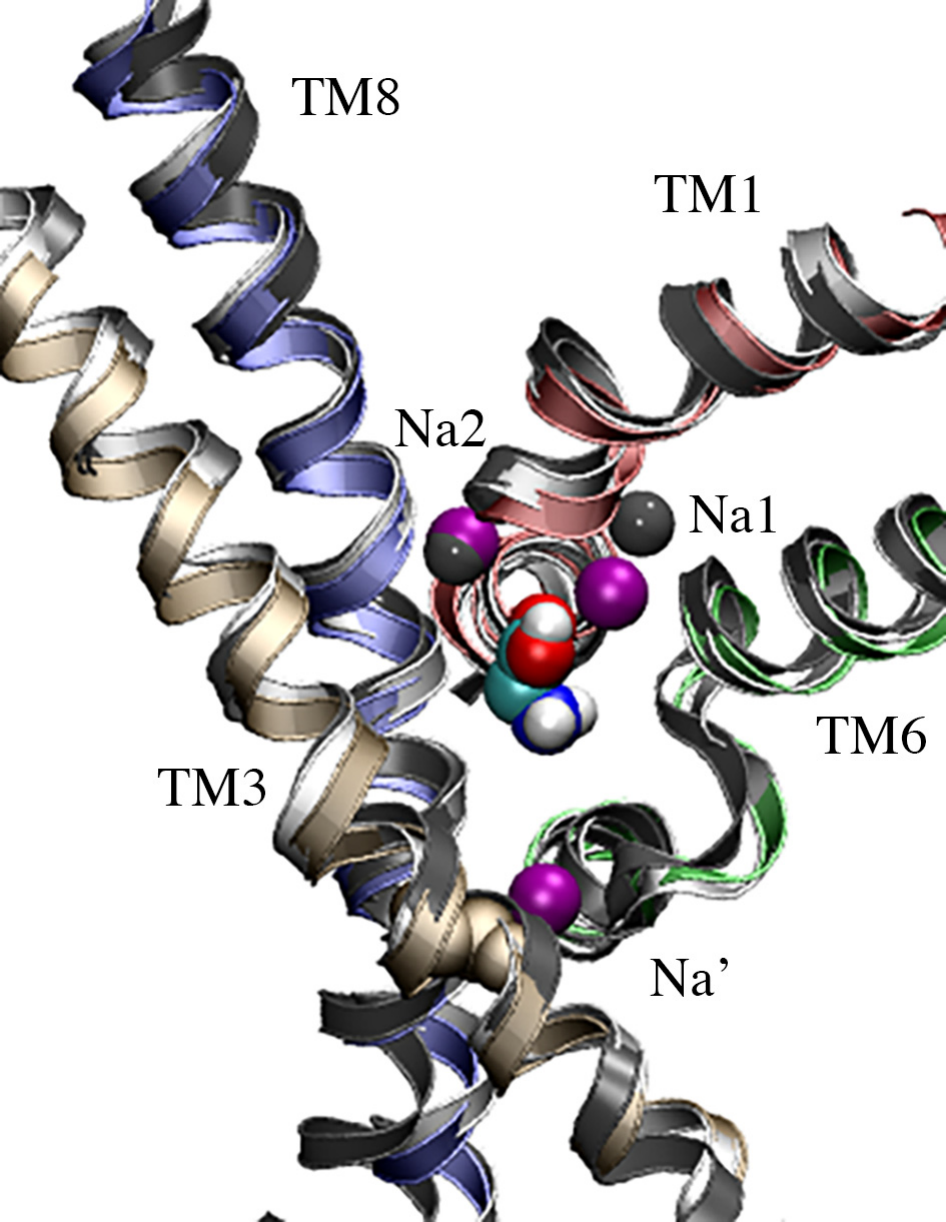
Comparison of the Na binding sites in the LeuT_Aa_ (dark grey) and dDaT (light grey) crystal structures and the GlyT2 model (colored helices). Crystallographic Na^+^ from the LeuT_Aa_ Na1 and Na2 sites are shown as dark grey spheres. The three modeled Na^+^ in GlyT2 are colored purple. Na^+^ occupies the Na1 and Na2 sites, and the proposed Na’ site of GlYT2. The substrate glycine is shown in CPK spacefill.

Two Na^+^ ions were placed in the GlyT2 model in positions corresponding to the identified Na1 and Na2 binding sites of the dDAT (PDBid: 4M48) and LeuT_Aa_ (PDBid: 3TT1) crystal structures. Fig 1 shows the superposition of the Na1 and Na2 sites in dDAT, LeuT_Aa_ and GlyT2 model. The two Na^+^ ions were placed between the residues from TM helices 1, 6, 7 and 8 forming a caged conformation, as shown in Fig 1. A third Na^+^ ion was placed in close proximity to residues S280 and A284 (TM3), which correspond to the Na1' binding site residues of BetP. The substrate glycine was placed in its binding site, coordinated by Na1. The final model of GlyT2 containing 3 Na^+^ ions and the substrate glycine was embedded in a pre-equilibrated POPC bilayer [28] downloaded from ATB [29]. The system was solvated in a water box and 150mM of NaCl was added to the system to mimic the experimental conditions. Counter-ions were added to maintain the overall charge neutrality of the system.

To initiate the simulation, 1000 steps of steepest descent energy minimization was performed. Then the system was equilibrated over a period of 10 ns, in which the position restraints were gradually lowered from 1000 kJ**·**mol^-1^**·**nm^-2^ to 500 kJ**·**mol^-1^**·**nm^-2^ to 100 kJ**·**mol^-1^**·**nm^-2^ to 50 kJ**·**mol^-1^**·**nm^-2^ to 10 kJ**·**mol^-1^**·**nm^-2^ over successive 2 ns simulations. The system was further equilibrated for 10ns with minimal (10 kJ**·**mol^-1^**·**nm^-2^) position restraints on the non-hydrogen atoms to stabilize the backbone fluctuations often associated with homology models [30]. Finally, the system was then simulated for a further 50 ns without restraints. Five independent simulations with different sets of starting velocities were performed starting from the same configuration.

### Analysis

#### Root mean squared deviation (RMSD

To compare the configurations obtained from the MD simulation trajectories to the starting structure (homology model after energy minimization), the root mean square deviation (RMSD) was calculated using the method of Maiorov and Crippin [31] after performing a least square fit of each frame of the trajectory to the reference structure.

#### Protein contact residues / contacting residues

All the protein residues for which the average distance between the CA atoms lay within a 4.0 Å radius of the center of any atom of the glycine substrate or Na^+^ ions were considered to be in direct contact. In all cases, the averaging was performed over the five independent 50 ns simulations, giving a total cumulative simulation time of 250 ns.

#### Ion-protein and substrate-protein distances

To calculate the ion-protein and substrate-protein distances, the minimum distance between the center of mass of the glycine substrate or Na^+^ ion and center of any atom of the contacting residues were measured using the g_mindist program. In all cases except for the Na^+^ ion occupying the Na3 site, these distances were averaged over the cumulative 250 ns of MD simulation (5 x 50 ns). In the case of the Na^+^ ion occupying the proposed Na3 site, these distances were averaged over the last 35 ns of each of the five 50 ns simulations.

### Functional analysis of GlyT2 mutants in Xenopus laevis oocytes

GlyT2 was subcloned into the plasmid oocyte transcription vector (pOTV) [32]. Site-directed mutagenesis was performed using a PCR-based protocol as described previously [33] and all mutations were sequenced on both strands by Dye Terminator Cycle Sequencing (ABI PRISM, Perkin Elmer). The wild type and mutant transporter cDNAs were linearized with *Spe*I and cRNA transcribed with T7 RNA polymerase using the mMessage mMachine kit (Ambion Inc., TX, USA).

### Electrophysiology

All chemicals were obtained from Sigma unless otherwise stated. Stage V oocytes were surgically harvested from *Xenopus laevis* whilst under anaesthesia as described previously [33]. After removal of oocytes, the incision was stitched and the frog allowed to recover in isolation. All surgical procedures were approved by the University of Sydney Animal Ethics Committee (Protocol #5269) under the Australian Code of Practice for the Care and Use of Animals for Scientific Purposes. 20 ng of cRNA was injected into oocytes and incubated in standard frog ringers buffer (96 mM NaCl, 2 mM KCl, 1 Mm MgCl_2_, 1.8 mM CaCl_2_, 5 mM HEPES, pH7.5) supplemented with 50 μg/ml gentamycin, 2.5 mM sodium pyruvate and 0.5 mM theophylline at 16–18°C.

Two to four days after microinjection, current recordings from oocytes voltage-clamped at -60 mV were made using the two electrode voltage clamp technique with a Geneclamp 500 amplifier (Axon Instruments, Foster City, CA) interfaced with a MacLab 2e chart recorder (ADI Instruments, Sydney, Australia) using the chart software.

Recording solution for substrate concentration responses was normal frog Ringer’s solution. For Na^+^ titrations, NMDG^+^ was used as the substitute cation, and total cation concentration was 150 mM. Current (*I*) as a function of substrate concentration was fitted by least-squares analysis to a derivation of the Michaelis-Menten equation,

$$I=I_{max}\cdot \frac{\left[substrate\right]}{\left([substrate]+{EC}_{50}\right)}$$

where *I*max is the maximum current generated and EC_50_ is the substrate concentration which generates a half-maximal response. Na^+^ concentration responses were fit to the Hill equation,

$$\frac{I}{I_{max}}=\frac{{\left[substrate\right]}^{n}}{{\left[substrate\right]}^{n}+{\left({EC}_{50}\right)}^{n}}$$

where n is the Hill coefficient and all other terms are as described above.

## Results

### Model Validation

**Fig 2:**
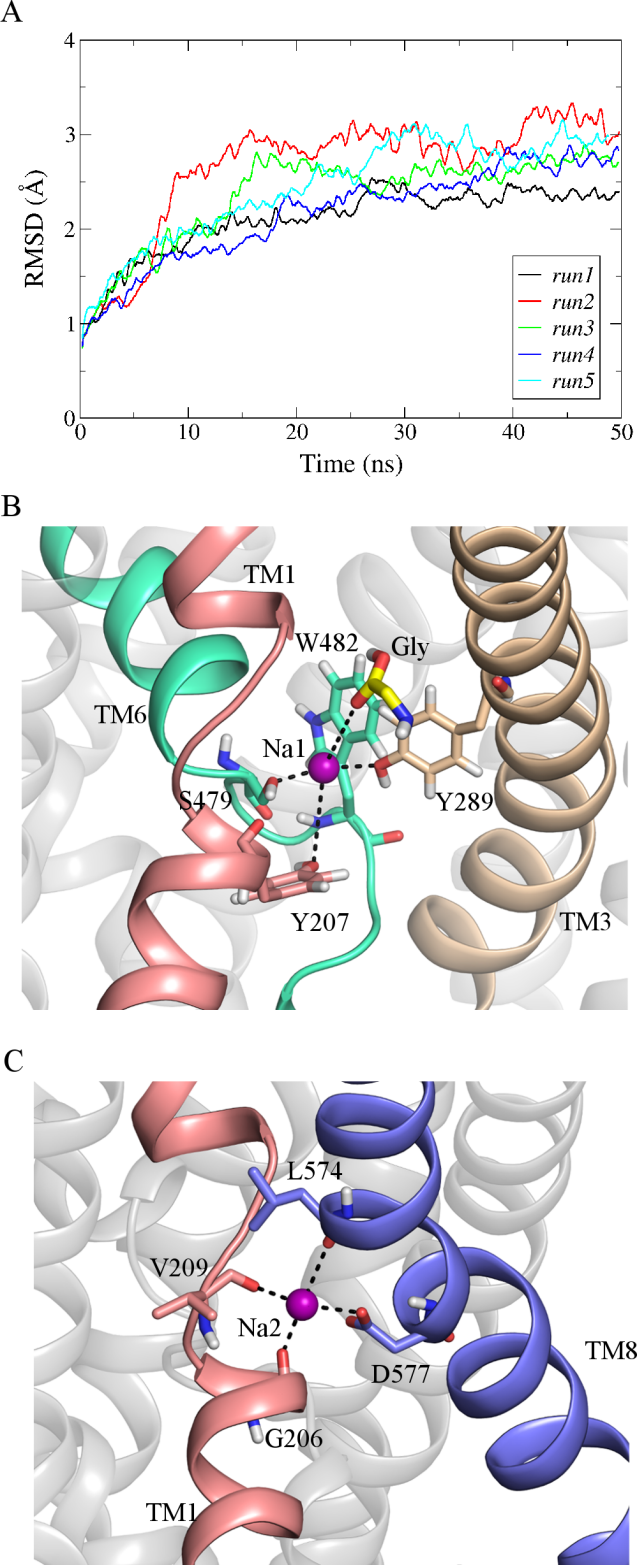
GlyT2 model stability and validation. (A) Backbone RMSD of GlyT2 model over 50 ns for all 5 runs. (B) and (C) shows the Na1 and Na2 binding sites in the GlyT2 model. GlyT2 is shown in a cartoon representation. The residues that bind Na^+^ (purple spacefill) are shown in licorice representation.

The backbone root mean square deviation (RMSD) between the GlyT2 homology model and that of the dDAT template structure (PDBid 4M48) was 1.37 Å. The initial model was evaluated using PROCHECK [34]. Analysis of the backbone geometry of the initial, non-refined homology model showed that 97% of residues lay in acceptable regions of the Ramachandran plot, lending confidence to geometry of the initial GlyT2 homology model. It should be noted that the refinement of protein models to reproduce experimental accuracy is still an ongoing challenge in structural bioinformatics [35, 36]. In the past decade, biased and non-biased MD simulation techniques of individual models and ensembles have proved a useful tool in model refinement on timescales of tens to hundreds of nanoseconds [37–39]. However, recent long-timescale simulations indicate that homology models drift significantly from the corresponding crystallographic structures on μs timescales [40]. To ensure the structural stability of the equilibrated GlyT2 model, unrestrained simulations were limited to 50 ns. Fig 2A shows the backbone RMSD of the modeled GlyT2 TM helices is stable across the five simulations, plateauing after 20 ns of simulation. The backbone RMSD, averaged across the last 30 ns of the five independent simulations, was 2.7 ± 0.3 Å. This RMSD is within the expected thermal fluctuations of a protein of this size [41].

In all simulations, the Na^+^ ions occupying both the Na1 and Na2 sites of GlyT2 formed stable interactions with the adjacent residues. The ion occupying the Na1 site diffused slightly within its binding site, coordinated by the side chain hydroxyl group of Y289 (TM3) and S479 (TM6) as shown in Fig 2B. W482 also contributes to the Na1 site via a cation-π interaction with the resident Na^+^ (Fig 2B). In addition, this Na^+^ also makes transient contacts with the side chain hydroxyl groups of Y207 (TM1), T578 (TM8) and the glycine substrate. During the simulations, the Na^+^ ion occupying the Na2 site is coordinated by the backbone interactions from the residues G206, V209 (TM1) and L574 (TM8) as shown in Fig 2C. The side chain carboxyl group of D577 (TM8) further stabilizes the Na^+^ in its binding site, as previously reported [12]. These interactions are similar to those observed in the crystal structures of dDAT and LeuT_Aa_. The distance between the resident Na^+^ ions and the surrounding residues of the Na1 and Na2 sites averaged over all five MD simulations, are provided as Supporting Information (Tables A and B in S1 File).

Prior to equilibration, the substrate glycine was placed at its binding site, in the vicinity of the predicted binding residues I283 (TM3), S479 (TM6), W482 (TM6), T578 (TM8) and T582 (TM8). During unrestrained MD simulations, glycine remained within the S1 site, coordinated primarily by T578 (TM8) and W482 (TM6). As glycine was unrestrained, and thus free to move in the simulations, its position and orientation varied within the S1 site throughout the simulations. The substrate glycine also formed direct contacts (within a 4.0 Å radius) with residues adjacent to the S1 binding site residues, namely Y287 (TM3), W215 (TM1), Y286 (TM3) and G575 (TM8) throughout the simulations. These interactions persisted for >80% of the time, averaged across the five independent simulations. It should be noted that some fluctuations or variations in the set of interacting residues are expected in MD simulations on timescales of tens or hundreds of ns, in which neither the ion or protein is restrained to a predetermined set of coordinates. The distance between the substrate glycine and the interacting residues, averaged over all five MD simulations, are provided as Supporting Information (Table C in S1 File).

**Fig 3:**
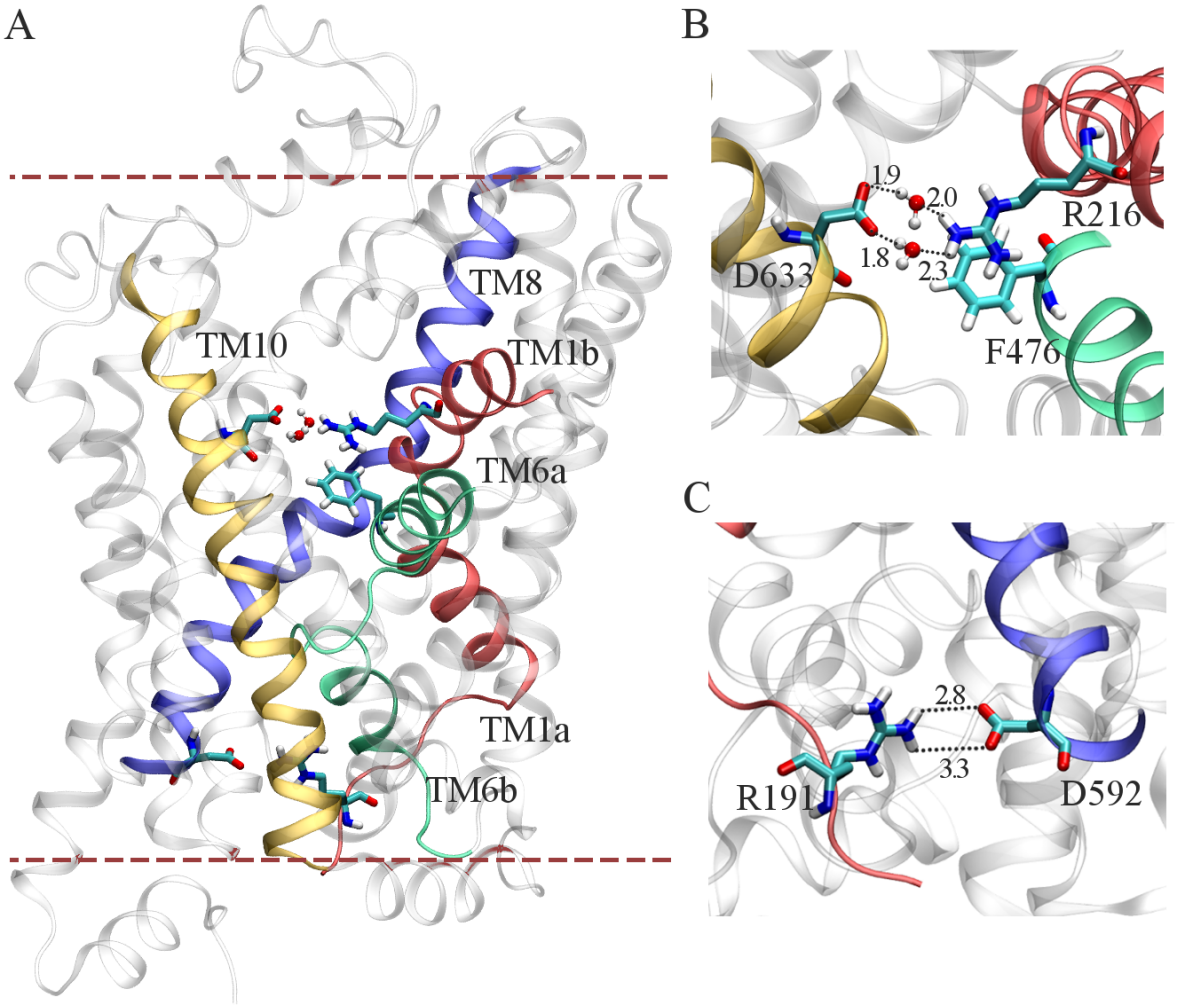
Initial conformation of the membrane-embedded, equilibrated GlyT2 model in MD simulations. (A) Outward occluded conformation of the GlyT2 model for MD simulation. (B) Water mediated salt bridges between R216 (TM1b) and D633 (TM10) and R216 forms a cation-π interaction with F476 (TM6a). (C) R191 from TM1 and D592 from TM8 form a salt bridge.

The Na^+^/Cl^-^ dependent family of transporter proteins undergo conformational change from an outward-open state to an inward open-state during the transport cycle [10, 42]. These transporters also adopt an intermediate substrate bound conformation known as the outward occluded state [42]. In order to validate that the modeled outward occluded conformation of the substrate bound GlyT2 model (shown in Fig 3A) is consistent with the experimentally characterized outward occluded conformation, the interactions between the conserved residue pairs that form the extracellular (R216 (TM1b) / D633 (TM10)) and intracellular gates (R191 (TM1a) / D592 (TM8)) were examined.

Fig 3B shows the residues R216 and D633 form a water mediated salt bridge and also the residues R216 and F476 (TM6a) form a cation-π interaction between their respective guanidinium and phenyl groups. In addition to these, Fig 3C shows the direct contact between the residues R191 and D592 on the intracellular side of the protein. The interactions identified in the membrane embedded GlyT2 model are in agreement with the previously observed conformations of Na^+^/Cl^-^ dependent transporters in the outward occluded state [10, 42] and demonstrate that the model reproduces an essential feature of all SLC6 transporters.

### Characterizing a novel Na3 binding site in GlyT2

**Fig 4:**
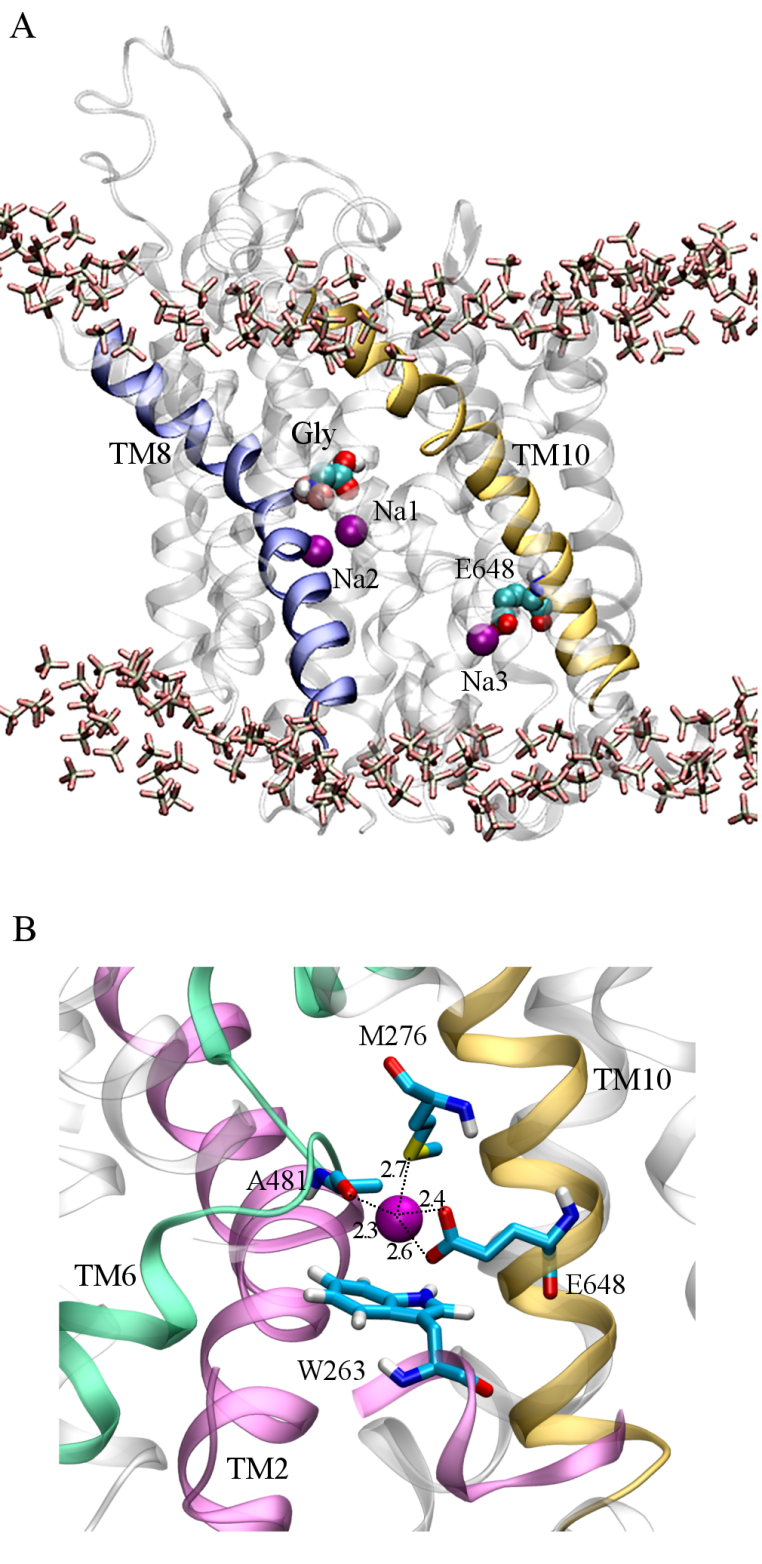
Location of the proposed Na3 site in GlyT2. (A) Final conformation of GlyT2 after 50 ns unrestrained MD simulation. Three Na^+^ ions (purple spacefill) remain stably bound to GlyT2, occupying the Na1 and Na2 sites, and a third site, Na3, where E648 (CPK spacefill) interacts electrostatically with the Na^+^ ion. The substrate glycine is shown in CPK spacefill and the membrane headgroups are in licorice representation. (B) A close-up view of the Na3 site. The residues that form the Na3 site are shown in CPK.

**Fig 5:**
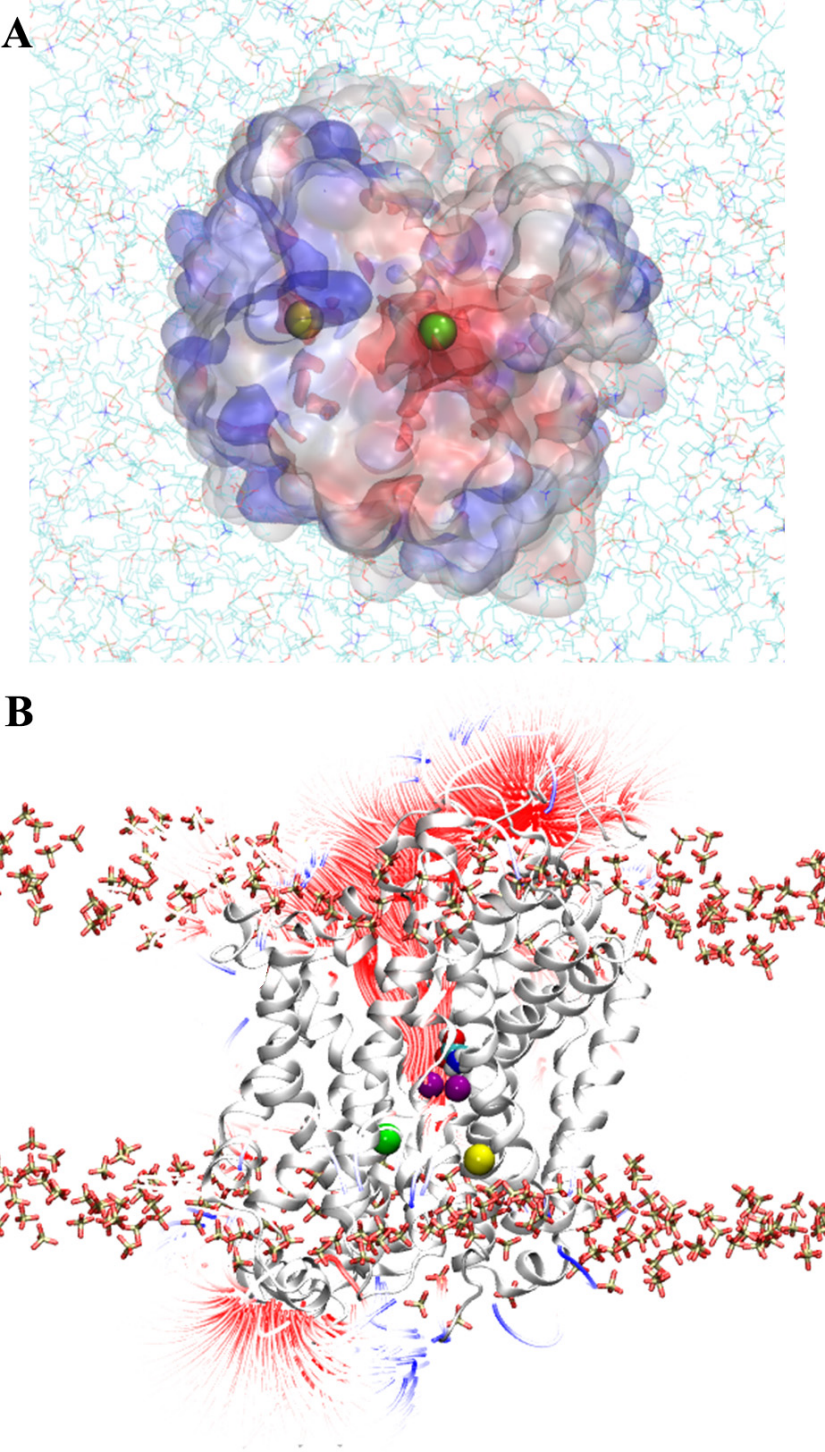
Electrostatic potential of the GlyT2 model. The electrostatic potential, contoured at +2 and -2 kT/e, was calculated at pH 7.0 using 150 mM NaCl and a relative dielectric permittivity of 78.5. Negative potential is red, positive is blue. (A) Electrostatic potential map of the intracellular surface of GlyT2, viewed from the intracellular side, normal to the membrane. Membrane phospholipids are shown as CPK colored lines. The initial placement of the third Na^+^ is in yellow. The final position of Na^+^ from MD simulations, bound to the proposed Na3 site, is in green. (B) Side view of the GlyT2 model, from the plane of the membrane, shows the density of the electric field lines. The protein is in gray cartoon representation and the substrate glycine is in CPK spacefill representation. Na^+^ ions occupying the Na1 and Na2 sites are purple.

To identify and characterize the Na3 binding site of GlyT2, a third Na^+^ was initially placed at the location consistent with the Na1' site of BetP, close to S280 and A284 (TM3), as shown in Fig 1. In all five simulations, this Na^+^ spontaneously dissociated from its initial location within the first 10 ns of simulation and diffused along a water-filled cleft in the protein to bind to a previously uncharacterized site in GlyT2, shown in Fig 4A, where it interacts electrostatically with E648 (TM10). It should be noted that E648 is solvated and is accessible from the intracellular solution (Fig B in S1 File). Based on solvent accessibility and pKa predictions, E648 was predicted to carry a negative charge at physiological pH [18, 19]. In addition to E648, the binding of Na^+^ was coordinated by residues W263, M276 (TM3) and A481 (TM6), as shown in Fig 4B. The spontaneous binding of Na^+^ to this site, referred to as the Na3 site, persisted for the duration of all five simulations. Na^+^ binding at the Na3 site is dominated by the electrostatic interactions with the carboxylate side chain of E648, and is further stabilized by interactions with the backbone carbonyl of A481, a cation-π interaction with W263 and the M276 S-methyl thioether side chain, as shown in Fig 4B. Note that W263 lies in a loop region immediately preceding TM3. The orientation of the loop, and W263, fluctuates throughout the simulations prior to Na^+^ binding. The binding of Na^+^ at the proposed Na3 site stabilises the orientation of W263, effectively blocking the exit of Na^+^ from the Na3 site. The distance between the Na^+^ ion and the contacting residues forming the new Na3 site, averaged over the last 35ns of the five independent MD simulations are provided as Supporting Information (Table D in S1 File). Mapping of the electrostatic potential [43] of GlyT2 demonstrated that solvent accessible surface of the GlyT2 homology model in the region surrounding residues S280 and A284 was largely electropositive, as shown in Fig 5A, and thus, is not expected to provide an electrostatically favorable environment for a Na^+^ binding site in GlyT2. In contrast, the GlyT2 model contained large electronegative region in the vicinity of the Na3 binding site, stabilizing the binding of Na^+^. It should also be noted that in one of the MD simulations, a Na^+^ ion diffused into the transporter from the intracellular solution. Fig 5B shows the electric field lines (contoured at ± 2 kT/e) arising from the electrostatic potential. In the outward occluded conformation of the GlyT2 model, two primary regions of electronegativity (red field lines) funnel Na^+^ towards the Na1 and Na3 binding sites. The GlyT2 homology model maps the membrane-embedded regions of the protein, and assumes the GlyT2 structure is homologous to that of dDAT. Furthermore, electrostatic effects arising from regions of the protein not contained within the model cannot be accounted for in this study.

### Role of E648 in forming Na3 site in GlyT2

In order to validate the role of E648 in Na^+^ binding at the proposed Na3 site via MD simulation, the E648 (TM10) of the GlyT2 comparative model was mutated to methionine. The GlyT2 E648M mutation was generated for two reasons. First, the mutation removes the critical negative charge required for stable Na^+^ binding at Na3 identified in the MD simulations. Second, a methionine residue is found at the corresponding position for GlyT1, which is coupled to the co-transport of only 2 Na^+^ ions. To initiate the simulations, the substrate glycine was placed in its binding site and Na^+^ ions were placed at the Na1 and Na2 sites, and at the proposed Na3 site identified above. The system was equilibrated and 50 ns of unrestrained MD simulation was performed. On mutation of E648 to methionine, Na^+^ diffused out of the proposed Na3 site within the first 15 ns of simulation.

### Functional Analysis of GlyT2 Mutants

The study by Perez-Siles *et al*. [12] demonstrated that mutations of residues that form the Na1 and Na2 sites of GlyT2 produce transporters with increased *K*_0.5_ values for both glycine and Na^+^ compared to WT. We repeated some of these mutations (S479G (Na1) and L574I (Na2) in GlyT2) and observed similar results to that of Perez-Siles and co-workers (Table 1).

**Table 1:** Glycine and Na^+^ K_0.5_ values and Hill coefficients for WT and GlyT2 mutants.

Site	Mutant	K_0.5_ (glycine, μM)	K_0.5_ (Na+, mM)	Hill Co-efficient (Na+)
	WT	18.2 ± 0.6	33 ± 1	2.5 ± 0.2
Na1	S479G	84 ± 12	43 ± 6	3.5 ± 1.0
Na2	L574I	17 ± 1	49 ± 4	1.9 ± 0.1
Na3	E248A	9 ± 1	32 ± 18	1.8 ± 0.4
Na3	E648M	8.5 ± 2.8	>100	ND
Na3	M276A	32 ± 3	40 ± 6	2.5 ± 0.6
Na3	W263L	ND	ND	

Data shown represents the mean ± SEM, n ≥ 4. Glycine application to oocytes expressing the W263L mutant did not generate currents and it was not possible to measure glycine and Na^+^ K_0.5_ values.

The changes in function associated with these mutations together with the MD simulations and the identification of Na^+^ bound to the crystal structures of LeuT_Aa_ and dDAT suggest that the Na1 and Na2 sites are conserved in GlyT2.

**Fig 6:**
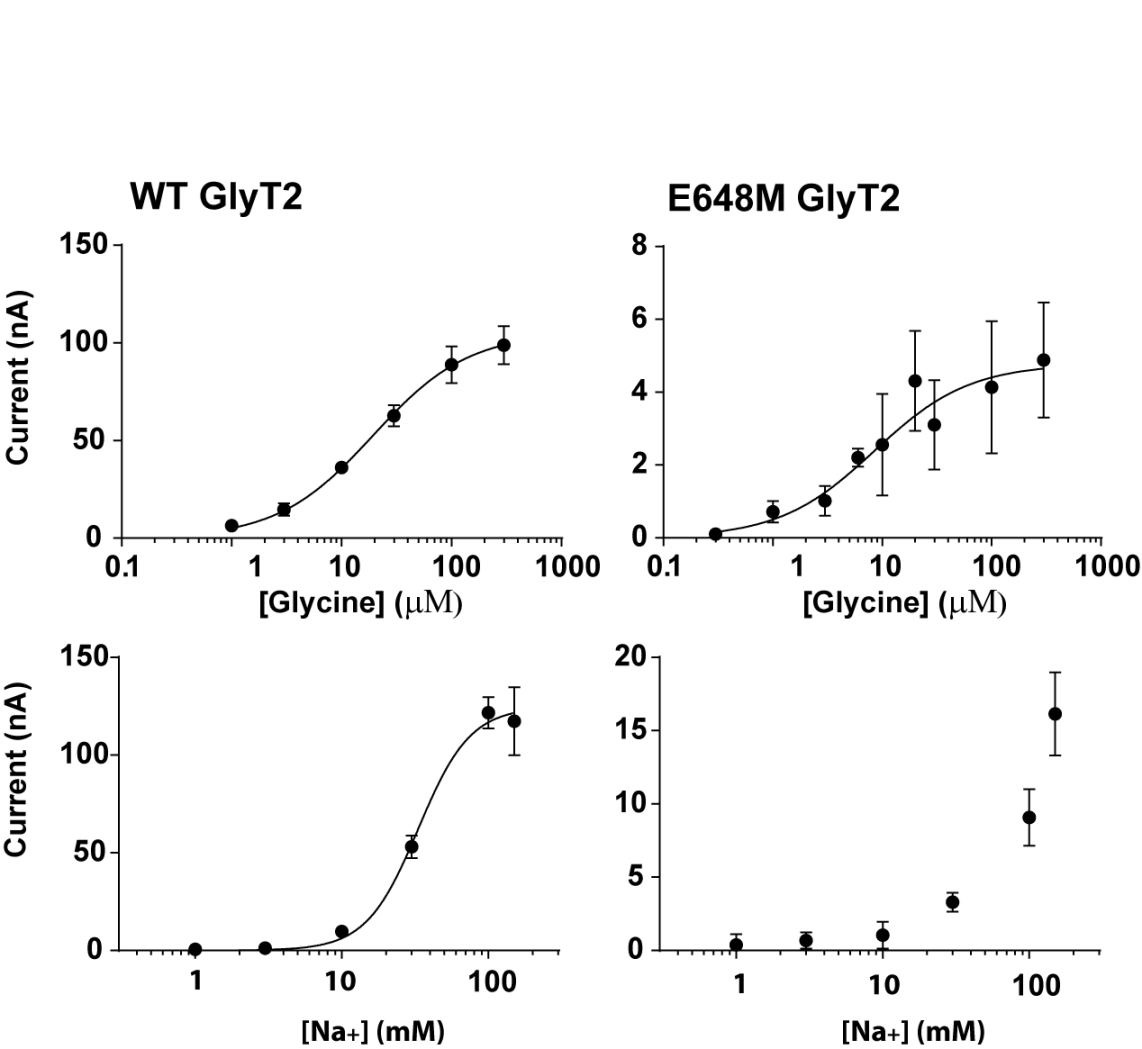
Glycine (top panels) and Na^+^ (bottom panel) concentration-dependent transport currents mediated by WT GlyT2 and GlyT1 and the Na3 site mutant in GlyT2, E648M mutant. Glycine concentration-dependent transport currents were measured in ND96 and Na^+^ concentration-dependent transport currents were measured in the presence of an EC_90_ concentration of glycine for the respective transporters. Currents were normalized to the maximal transport current in each case except for the Na^+^ concentration dependent currents for the E648M mutant because the currents did not appear to saturate at a maximal Na^+^ concentration.

To test the MD predictions of the Na3 site we generated mutations in the cDNA corresponding to the proposed Na3 site of GlyT2 and compared the glycine and Na^+^ concentration dependent transport currents of the mutants to corresponding values for wild type GlyT2. Three mutant GlyT2 transporters were produced incorporating either the W263L, M276A or E648M mutations. Glycine concentration responses were measured in the presence of 98.5 mM Na^+^ and Na^+^ concentration responses were measured in the presence of the EC_90_ concentration of glycine for each transporter. For wild type GlyT2, the EC_50_ for glycine is 19.4 ± 1.9 μM and the EC_50_ for Na^+^ is 33 ± 1 mM, with a Hill co-efficient of 2.5 ± 0.2 (Fig 6). The maximal currents observed for WT transporters is generally in the range of 100–200 nA depending on the batch of oocytes used. However, for the E648M mutant, expression levels were reduced compared to wild type, which is reflected in the maximal current amplitude achieved with 300 μM glycine being only 4.8 ± 0.4 nA (Fig 6). Despite the small amplitudes of glycine-evoked currents for E648M, it was possible to measure an EC_50_ for glycine of 8.5 ± 2.8 μM, which is not significantly different to that of WT GlyT2. However, the mutation did cause a substantial change in Na^+^ sensitivity. The Na^+^ concentration response did not saturate at concentrations up to 150 mM (Fig 6D) and it was not possible to use higher concentrations of Na^+^ because of the instability of the voltage clamp at higher Na^+^ concentrations. Nevertheless, these observations suggest that Na^+^, but not glycine, interactions have been compromised in the E648M mutant. We also investigated the functional impact of mutating the other two residues that coordinate Na3. For GlyT2 M276A, both the glycine and Na^+^ affinities, as well as the Hill co-efficient for Na^+^ were not substantially different to wild type (Table 1) suggesting that the mutation does not disrupt Na^+^ interactions with this site. Incorporation of the W263L mutation produced transporters with greatly reduced glycine-evoked currents, preventing characterization of the substrate and Na^+^ affinities.

## Discussion

GlyT2 is unique among the SLC6 family of transporters in that substrate transport is coupled to the co-transport of 3 Na^+^ ions. The starting hypothesis of this study was that the Na3 site of GlyT2 corresponds to the Na1' site of BetP. However, in all 5 of the MD simulations the Na^+^ initially placed in this site was not stably coordinated and diffused to an alternate Na3 binding site, where it was stably coordinated by the side chains of E648 (TM10), W263 (TM3) and M276 (TM3) and backbone interactions with A481 (TM6) (Fig 4B) for the remainder of all five independent simulations. It is of particular note that the Na3 binding site characterized here is spatially well separated from the location corresponding to the BetP Na1' site and also the Na1 and Na2 sites. Furthermore, the E648 residue is not conserved between GlyT2 and the closely related GlyT1 transporter, and as such, this site is unlikely to form a Na^+^ ion binding site in GlyT1. The GlyT2 E648M mutant displayed reduced maximal glycine-evoked currents, without changing the EC_50_ for glycine, and also reduced apparent affinity for Na^+^. This suggests that this novel site plays an important role in the transport mechanism. However, despite these clear disruptions to overall function of the GlyT2 transporter by the E648M mutation, there are a number of unresolved questions arising from this study. The proposed Na3 site is located towards the intracellular surface of the transporter and it is unlikely that this binding site will be the initial binding site for any of the three Na^+^ ions. In order for Na^+^ to reach this site from the external solution, it is likely to have to interact with other sites before reaching this location. In BetP the Na1' site is presumably accessible from the external solution and, if a similar access pathway exists in GlyT2, then the Na^+^ ion may diffuse into the equivalent site of the BetP Na1' site and then to the novel Na3 binding site identified in this study. A second possibility is that the novel Na3 site may be part of the permeation pathway of either of the Na^+^ ions bound to Na1 or Na2. Finally, in the inward facing structure of LeuT_Aa_ (PDBid: 3TT3) [10], there is an aqueous pathway from the region that corresponds to the proposed Na3 site of GlyT2 to the intracellular solution. Indeed, a Na^+^ ion was observed to access the novel Na3 site from the intracellular solution in one MD simulation. Throughout all simulations, E648 is solvated and accessible from the intracellular solution. Therefore, it is possible that Na^+^ may enter the transporter from the intracellular side and binding of Na^+^ to the novel site may facilitate transport process rather than being the 3^rd^ Na^+^ coupling ion.

This study has provided evidence that identifies a novel Na^+^ binding site on GlyT2 that influences the function of the transporter. At this stage it remains to be seen whether this novel site represents the binding site for the 3^rd^ Na^+^ ion required for transport by GlyT2. Whilst it would be desirable to conduct longer simulations to begin to address some of these issues, the use of a homology model of GlyT2 based on dDAT that has only 50% amino acid sequence identity has the potential to introduce substantial errors and as such alternate approaches will be required.

## Supporting Information

S1 File Multiple sequence alignment of GlyT2 and its homologues, and distance information for coordinating residues in each of the Na binding sites. (PDF)

S2 File Initial GlyT2 homology model, prior to MD equilibration. (PDB)

S3 File Membrane-embedded GlyT2 homology model with bound Na^+^ and substrate glycine. Taken from Run 1 of 5 MD production runs, at time = 41 ns.
(PDB)

S4 File Membrane-embedded GlyT2 homology model with bound Na^+^ and substrate glycine. Taken from Run 2 of 5 MD production runs, at time = 48 ns.
(PDB)

## References

[1] Roux MJ, Supplisson S. Neuronal and glial glycine transporters have different stoichiometries. Neuron. 2000;25:373–83. 10719892 10.1016/s0896-6273(00)80901-0

[2] Eulenburg V, Armsen W, Betz H, Gomeza J. Glycine transporters: essential regulators of neurotransmission. Trends in Biochemical Sciences. 2005;30:325–33. 15950877 10.1016/j.tibs.2005.04.004

[3] Harvey RJ, Yee BK. Glycine transporters as novel therapeutic targets in schizophrenia, alcohol dependence and pain. Nature Reviews Drug Discovery. 2013;12:866–85. 10.1038/nrd3893 24172334

[4] Dohi T, Morita K, Kitayama T, Motoyama N, Morioka N. Glycine transporter inhibitors as a novel drug discovery strategy for neuropathic pain. Pharmacology & Therapeutics. 2009;123:54–79. 19393690 10.1016/j.pharmthera.2009.03.018

[5] Beuming T, Shi L, Javitch JA, Weinstein H. A comprehensive structure-based alignment of prokaryotic and eukaryotic neurotransmitter/Na+ symporters (NSS) aids in the use of the LeuT structure to probe NSS structure and function. Molecular Pharmacology. 2006;70:1630–42. 16880288 10.1124/mol.106.026120

[6] Kristensen AS, Andersen J, Jorgensen TN, Sorensen L, Eriksen J, Loland CJ, et al SLC6 neurotransmitter transporters: structure, function, and regulation. Pharmacological Reviews. 2011;63:585–640. 10.1124/pr.108.000869 21752877

[7] Attwell D, Barbour B, Szatkowski M. Nonvesicular release of neurotransmitter. Neuron. 1993;11:401–7. 8104430 10.1016/0896-6273(93)90145-h

[8] Supplisson S, Roux MJ. Why glycine transporters have different stoichiometries. FEBS Letters. 2002;529:93–101. 12354619 10.1016/s0014-5793(02)03251-9

[9] Gomeza J, Ohno K, Hulsmann S, Armsen W, Eulenburg V, Richter DW, et al Deletion of the mouse glycine transporter 2 results in a hyperekplexia phenotype and postnatal lethality. Neuron. 2003;40:797–806. 14622583 10.1016/s0896-6273(03)00673-1

[10] Krishnamurthy H, Gouaux E. X-ray structures of LeuT in substrate-free outward-open and apo inward-open states. Nature. 2012;481:469–74. 10.1038/nature10737 22230955 PMC3306218

[11] Penmatsa A, Wang KH, Gouaux E. X-ray structures of Drosophila dopamine transporter in complex with nisoxetine and reboxetine. Nature Structural & Molecular Biology. 2015;22:506–08. 10.1038/nsmb.3029 PMC4608549 25961798

[12] Perez-Siles G, Nunez E, Morreale A, Jimenez E, Leo-Macias A, Pita G, et al An aspartate residue in the external vestibule of GLYT2 (glycine transporter 2) controls cation access and transport coupling. Biochemical Journal. 2012;442:323–34. 10.1042/BJ20110247 22132725

[13] Ressl S, Terwisscha van Scheltinga AC, Vonrhein C, Ott V, Ziegler C. Molecular basis of transport and regulation in the Na(+)/betaine symporter BetP. Nature. 2009;458:47–52. 10.1038/nature07819 19262666

[14] Khafizov K, Perez C, Koshy C, Quick M, Fendler K, Ziegler C, et al Investigation of the sodium-binding sites in the sodium-coupled betaine transporter BetP. Procedings of the National Academy of Sciences USA. 2012;109:E3035–44. 10.1073/pnas.1209039109 PMC3497817 23047697

[15] Perez C, Faust B, Mehdipour AR, Francesconi KA, Forrest LR, Ziegler C. Substrate-bound outward-open state of the betaine transporter BetP provides insights into Na+ coupling. Nature Communications. 2014;5:4231 10.1038/ncomms5231 25023443 PMC4745115

[16] Kelley LA, Sternberg MJE. Protein structure prediction on the Web: a case study using the Phyre server. Nature Protocols. 2009;4:363–71. 10.1038/nprot.2009.2 19247286

[17] Yamashita A, Singh SK, Kawate T, Jin Y, Gouaux E. Crystal structure of a bacterial homologue of Na+/Cl—dependent neurotransmitter transporters. Nature. 2005;437:215–23. 16041361 10.1038/nature03978

[18] Li H, Robertson AD, Jensen JH. Very fast empirical prediction and rationalization of protein pKa values. Proteins: Structure, Function, and Bioinformatics. 2005;61:704–21. 10.1002/prot.20660 16231289

[19] Bas DC, Rogers DM, Jensen JH. Very fast prediction and rationalization of pKa values for protein-ligand complexes. Proteins: Structure, Function, and Bioinformatics. 2008;73:765–83. 10.1002/prot.22102 18498103

[20] van der Spoel D, Lindahl E, Hess B, Groenhof G, Mark AE, Berendsen HJC. GROMACS: fast, flexible, and free. Journal of Computational Chemistry. 2005;26:1701–18. 16211538 10.1002/jcc.20291

[21] Schmid N, Eichenberger AP, Choutko A, Riniker S, Winger M, Mark AE, et al Definition and testing of the GROMOS force-field versions 54A7 and 54B7. European Biophysics Journal. 2011;40:843–56. 10.1007/s00249-011-0700-9 21533652

[22] Hermans J, Berendsen HJC, van Gunsteren WF, Postma JPM. A consistent empirical potential for water-protein interactions. Biopolymers. 1984;23:1513–18.

[23] Berendsen HJC, Postma JPM, van Gunsteren WF, Dinola A, Haak JR. Molecular-dynamics with coupling to an external bath. Journal of Chemical Physics. 1984;81:3684–90.

[24] Hess B, Bekker H, Berendsen HJC, Fraaije JGEM. LINCS: A linear constraint solver for molecular simulations. Journal of Computational Chemistry. 1997;18:1463–72.

[25] Miyamoto S, Kollman PA. SETTLE: an analytical version of the shake and rattle algorithm for rigid water models. Journal of Computational Chemistry. 1992;13:952–62.

[26] Feenstra KA, Hess B, Berendsen HJC. Improving efficiency of large time-scale molecular dynamics simulations of hydrogen-rich systems. Journal of Computational Chemistry. 1999;20:786–98. 10.1002/(SICI)1096-987X(199906)20:8<786::AID-JCC5>3.0.CO;2-B 35619462

[27] Tironi IG, Sperb R, Smith PE, van Gunsteren WF. A generalized reaction field method for molecular dynamics simulations. Journal of Chemical Physics. 1995;102:5451–59.

[28] Poger D, van Gunsteren WF, Mark AE. A new force field for simulating phosphatidylcholine bilayers. Journal of Computational Chemistry. 2010;31:1117–25. 10.1002/jcc.21396 19827145

[29] Malde AK, Zuo L, Breeze M, Stroet M, Poger D, Nair PC, et al An automated force field topology builder (ATB) and repository: version 1.0. Journal of Chemical Theory and Computation. 2011;7:4026–37. 10.1021/ct200196m 26598349

[30] Fan H, Mark AE. Mimicking the action of folding chaperones in molecular dynamics simulations: Application to the refinement of homology-based protein structures. Protein Science. 2004;13:992–99. 15010545 10.1110/ps.03449904 PMC2280060

[31] Maiorov VN, Crippen GM. Significance of root-mean-square deviation in comparing three-dimensional structures of globular proteins. Journal of Molecular Biology. 1994;235:625–34. 10.1006/jmbi.1994.1017 8289285

[32] Wiles AL, Pearlman R-J, Rosvall M, Aubrey KR, Vandenberg RJ. N-Arachidonyl-glycine inhibits the glycine transporter, GLYT2a. Journal of Neurochemistry. 2006;99:781–86. 16899062 10.1111/j.1471-4159.2006.04107.x

[33] Vandenberg RJ, Shaddick K, Ju P. Molecular basis for substrate discrimination by glycine transporters. Journal of Biological Chemistry. 2007;282:14447–53. 17383967 10.1074/jbc.M609158200

[34] Laskowski R, MacArthur M, Moss D, Thornton J. PROCHECK—a program to check the stereochemical quality of protein structures. Journal of Applied Crystallography. 1993;26:283–91.

[35] Misura KMS, Baker D. Progress and challenges in high-resolution refinement of protein structure models. Proteins: Structure, Function, and Bioinformatics. 2005;59:15–29. 10.1002/prot.20376 15690346

[36] Moult J. A decade of CASP: progress, bottlenecks and prognosis in protein structure prediction. Current Opinion in Structural Biology. 2005;15:285–89. 15939584 10.1016/j.sbi.2005.05.011

[37] Chen J, Brooks CL. Can molecular dynamics simulations provide high-resolution refinement of protein structure? Proteins: Structure, Function, and Bioinformatics. 2007;67:922–30. 10.1002/prot.21345 17373704

[38] Fan H, Mark AE. Refinement of homology-based protein structures by molecular dynamics simulation techniques. Protein Science. 2004;13:211–20. 14691236 10.1110/ps.03381404 PMC2286528

[39] Zhu J, Fan H, Periole X, Honig B, Mark AE. Refining homology models by combining replica-exchange molecular dynamics and statistical potentials. Proteins: Structure, Function, and Bioinformatics. 2008;72:1171–88. 10.1002/prot.22005 PMC2761145 18338384

[40] Raval A, Piana S, Eastwood MP, Dror RO, Shaw DE. Refinement of protein structure homology models via long, all-atom molecular dynamics simulations. Proteins: Structure, Function, and Bioinformatics. 2012;80:2071–79. 10.1002/prot.24098 22513870

[41] Maiorov VN, Crippin GM. Significance of root-mean-square deviation in comparing three-dimensional structures of globular proteins. Journal of Molecular Biology. 1994;235:625–34. 8289285 10.1006/jmbi.1994.1017

[42] Boudker O, Ryan RM, Yernool D, Shimamoto K, Gouaux E. Coupling substrate and ion binding to extracellular gate of a sodium-dependent aspartate transporter. Nature. 2007;445:387–93. 17230192 10.1038/nature05455

[43] Baker NA, Sept D, Joseph S, Holst MJ, McCammon JA. Electrostatics of nanosystems: Application to microtubules and the ribosome. Proceedings of the National Academy of Sciences USA. 2001;98:10037–41. 10.1073/pnas.181342398 PMC56910 11517324

