# Correction: Identification of a 3^rd^ Na^+^ Binding Site of the Glycine Transporter, GlyT2

**DOI:** 10.1371/journal.pone.0159896

**Published:** 2016-07-19

**Authors:** Nandhitha Subramanian, Amanda J. Scopelliti, Jane E. Carland, Renae M. Ryan, Megan L. O’Mara, Robert J. Vandenberg

The second author’s name is spelled incorrectly. The correct name is: Amanda J. Scopelliti. The correct citation is: Subramanian N, Scopelliti AJ, Carland JE, Ryan RM, O’Mara ML, Vandenberg RJ (2016) Identification of a 3^rd^ Na^+^ Binding Site of the Glycine Transporter, GlyT2. PLoS ONE 11(6): e0157583. doi:10.1371/journal.pone.0157583
