# Supplementary material for: Identification of a 3rd Na+ Binding Site of the Glycine Transporter, GlyT2
Source: PLoS One. 2016 Jun 23;11(6):e0157583. doi: 10.1371/journal.pone.0157583 (PMC4919009; doi:10.1371/journal.pone.0157583)
Supplement: S1 File — (PDF) [file pone.0157583.s001.pdf]

# Supporting Information

# Identification of a 3rd $Na^+$ binding site of the glycine transporter, GlyT2

Nandhitha Subramanian[1], Amanda J. Scopelitti[2,3], Jane E. Carland[2], Renae M. Ryan[2], Megan L. O'Mara[1#] and Robert J. Vandenberg[2#]

[1] Research School of Chemistry, The Australian National University, Canberra, ACT, 2601, Australia

[2] Discipline of Pharmacology, School of Medical Sciences, University of Sydney, Sydney, NSW, 2006, Australia

[3] Department of Physiology and Biophysics, Weill Cornell Medical College, New York, NY 10021, USA

[#] To whom correspondence should be addressed:

Megan L. O'Mara, Research School of Chemistry, The Australian National University, Canberra, ACT, 2601, Australia, Email: megan.o'mara@anu.edu.au

Robert J. Vandenberg, Discipline of Pharmacology, Bosch Institute, School of Medical Sciences, University of Sydney, Sydney, NSW, 2006, Australia, Email: robert.vandenberg@sydney.edu.au

```
dDAT      -------------------------------------------------NS---ISDERE
hNET      MLLARMNPQVQPENNGADTGPEQPLRARKTAELLVVKERNGVQCLLAPRDG---DAQPRE
LeuT      ----------------------------------------------------------RE
GlyT2     ----------------------------------------SVATVATQEDEQGDENKARG
GlyT1     ----------------------------------------AVPSEATKRDQ----NLKRG
BetP      ------------------------LENPTNLEGKLA----DAEEEIILEGE---DTQASL

dDAT      TWS---GKVDFLLSVIGFAVDLANVW-RFPYLCY----KNGGGAFLV--PYGIMLAVGGI
hNET      TWG---KKIDFLLSVVGFAVDLANVW-RFPYLCY----KNGGGAFLI--PYTLFLIIAGM
LeuT      HWA---TRLGLILAMAGNAVGLGNFL-RFPVQAA----ENGGGAFMI--PYIIAFLLVGI
GlyT2     NWS---SKLDFILSMVGYAVGLGNVW-RFPYLAF----QNGGGAFLI--PYLMMLALAGL
GlyT1     NWG---NQIEFVLTSVGYAVGLGNVW-RFPYLCY----RNGGGAFMF--PYFIMLIFCGI
BetP      NWSVIVPALVIVLATVVWGIGFKDSFTNFASSALSAVVDNLGWAFILFGTVFVFFIVVIA

dDAT      PLFYMELALGQHNRKGAIT--------CWGRLVPLFKGIGYAVVL---------------
hNET      PLFYMELALGQYNREGAAT--------VW-KICPFFKGVGYAVIL---------------
LeuT      PLMWIEWAMGRYGGAQGHGTTPAIFYLLW--RNRFAKILGVFGLW---------------
GlyT2     PIFFLEVSLGQFASQGPVS--------VW-KAIPALQGCGIAMLI---------------
GlyT1     PLFFMELSFGQFASQGCLG--------VW-RISPMFKGVGYGMMV---------------
BetP      ASKFGTIRLGRIDEAPEFR---TV---SW-ISMMFAAGMGIDLMFYGTTEPLTFYRNGVP

dDAT      --------IAFYVDFYYNVIIAWSLRFFFASFTNSLPWTSCNNIWNTPNCR---------
hNET      --------IALYVGFYYNVIIAWSLYYLFSSFTLNLPWTDCGHTWNSPNCTDPK--LLNG
LeuT      --------IPLVVAIYYVYIESWTLGFAIKFLVGLVP-----EPPTDPDS------IL--
GlyT2     --------ISVLIAIYYNVIICYTLFYLFASFVSVLPWGSCNNPWNTPECKDKTKLLLDS
GlyT1     --------VSTYIGIYYNVVICIAFYYFFSSMTHVLPWAYCNNPWNTHDCAG----VLDA
BetP      GHDEHNVGVAMSTTMFHWTLHPWAIYAIVGLAIAYSTFRVGRKQLLSSAFVP----LIGE

dDAT      -------P-----------------------FESQGF--QSAASEYFNRYILELNRSEGI
hNET      SVLGNHTK-----------------------YSKYKF---TPAAEFYERGVLHLHESSGI
LeuT      ----------------------------------------RPFKEFLYSYI-------GV
GlyT2     CVISDHPKIQIKNSTFCMTAYPNVTMVNFTSQANKTF--VSGSEEYFKYFVLKI--SAGI
GlyT1     SNLTNGSR-------------PAALPSNLSHLLNHSLQRTSPSEEYWRLYVLKL--SDDI
BetP      KGAEGWLG----------------KLIDILAIIATVF----GTACSLGLGALQI--GAGL

dDAT      HDLGAIK----WDMALCLLIVYLICYFS----LWKGISTSGKVVWFTALFP--YAALLIL
hNET      HDIGLPQ----WQLLLCLMVVVIVLYFS----LWKGVKTSGKVVWITATLP--YFVLFVL
LeuT      PKGDEPI----LKPSLFAYIVFLITMFINVSILIRGI-SKGIERFAKIAMPTLFILAVFL
GlyT2     EYPGEIR----WPLALCLFLAWVIVYAS----LAKGIKTSGKVVYFTATFP--YVVLVIL
GlyT1     GNFGEVR----LPLLGCLGVSWLVVFLC----LIRGVKSSGKVVYFTATFP--YVVLTIL
BetP      SAANIIEDPSDWTIVGIVSVLTLAFIFS----AISGV-GKGIQYLSNANMV--LAALLAI

dDAT      LIRGL--------TLPG----SFLGIQYYLTPNFSAIYKAEV--------WADAATQVFF
hNET      LVHGV--------TLPG----ASNGINAYLHIDFYRLKEATV--------WIDAATQIFF
LeuT      VIRVF--------LLETPNGTAADGLNFLWTPDFEKLKDPGV--------WIAAVGQIFF
GlyT2     LIRGV--------TLPG----AGAGIWYFITPKWEKLTDATV--------WKDAATQIFF
GlyT1     FVRGV--------TLEG----AFDGIMYYLTPQWDKILEAKV--------WGDAASQIFY
BetP      FVFVVGPTVSILNLLPG-------SIGNYLSNFFQMAGRTAMSADGTAGEWLGSWTIFYW
```

```
dDAT      SLGPGFGVLLAYASYNKYHNNVYKDALLTSFINSATSFIAG--FVIFSVLGYMAHTLGVR
hNET      SLGAGFGVLIAFASYNKFDNNCYRDALLTSSINCITSFVSG--FAIFSILGYMAHEHKVN
LeuT      TLSLGFGAIITYASYVRKDQDIVLSGLTAATLNEKAEVILGGSISIPAAVAFFGVANAVA
GlyT2     SLSAAWGGLITLSSYNKFHNNCYRDTLIVTCTNSATSIFAG--FVIFSVIGFMANERKVN
GlyT1     SLGCAWGGLITMASYNKFHNNCYRDSVIISITNCATSVYAG--FVIFSILGFMANHLGVD
BetP      AWWISWSPFVGMFLARISRGRSIREFILGVLLVPAGVSTVW--FSIFGGTAIVFEQNGES

dDAT      IEDVATEGPGLVFVVYPAAIATMPASTFWALIFFMMLATLGLDSSFGGSEAIITALSDEF
hNET      IEDVATEGAGLVFILYPEAISTLSGSTFWAVVFFVMLLALGLDSSMGGMEAVITGLADDF
LeuT      IAKAGAFNLG--FITLPAIFSQTAGGTFLGFLWFFLLFFAGLTSSIAIMQPMIAFLEDEL
GlyT2     IENVADQGPGIAFVVYPEALTRLPLSPFWAIIFFLMLLTLGLDTMFATIETIVTSISDEF
GlyT1     VSRVADHGPGLAFVAYPEALTLLPISPLWSLLFFFMLILLGLGTQFCLLETLVTAIVDEV
BetP      I-----WGDGAAEEQLFGLLHALPGGQIMGIIAMILLGTFFITSA-DSASTVMGTMSQHG

dDAT      --PKIKRNRELFVAGLFSLYFVVGLASCTQGGFYFFHLLDRYAAGYSILVAVFFEAIAVS
hNET      --QVLKRHRKLFTFGVTFSTFLLALFCITKGGIYVLTLLDTFAAGTSILFAVLMEAIGVS
LeuT      --KLSRKHAVLWTAAIVFFSAHLVMFLNKS-----LDEMDFWAGTIGVVFFGLTELIIFF
GlyT2     -PKYLRTHKPVFTLGCCICFFIMGFPMITQGGIYMFQLVDTYAASYALVIIAIFELVGIS
GlyT1     GNEWILQKKTYVTLGVAVAGFLLGIPLTSQAGIYWLLLMDNYAASFSLVVISCIMCVAIM
BetP      ----QLEANKWVTAAWGVATAAIGLTLLLSGG----------------------------

dDAT      WIYGTNRFSEDIRD--MIGFPPGRYWQVCWRFVAPIFLLFITVYLLIGYEPLTYADYVYP
hNET      WFYGVDRFSNDIQQ--MMGFRPGLYWRLCWKFVSPAFLLFVVVVSIINFKPLTYDDYIFP
LeuT      WIFGADKAWEEINRGGIIKVPRIYYYVM--RYITPAFLAVLLVVWAREYIPKIMEETHWT
GlyT2     YVYGLQRFCEDIEM--MIGFQPNIFWKVCWAFVTPTILTFILCFSFYQWEPMTYGSYRYP
GlyT1     YIYGHRNYFQDIQM--MLGFPPPLFFQICWRFVSPAIIFFILVFTVIQYQPITYNHYQYP
BetP      -----DNALSNLQN---------------VTIVAATPFLFVVIGLMFALVKDLSNDVIYL

dDAT      SWANALGWCIAGSSVVMIPAVAIFKLLSTPG-SLRQRFTILTTPWRD--QQLVPR-----
hNET      PWANWVGWGIALSSMVLVPIYVIYKFLSTQG-SLWERLAYGITPENE--HHLVAQRDIRQ
LeuT      VW---------ITRFYIIGLFLFLTFLV----FLAERRRNHE------------------
GlyT2     NWSMVLGWLMLACSVIWIPIMFVIKMHLAPG-RFIERLKLVCSPQPDWGPFLAQHRGERY
GlyT1     GWAVAIGFLMALSSVLCIPLYAMFRLCRTDGDTLLQRLKNATKPSRDWGPALLEHRTGRY
BetP      EYREQQRFNARLARERRVHNEHRKRELAAK--RRRERKASGAGKRR--------------

dDAT      ----------------------------------------
hNET      FQLQHWLAI-------------------------------
LeuT      ----------------------------------------
GlyT2     KNMIDPLGTSSLGLK--------LPVKDLELGTQC-----
GlyT1     APTIAPSPEDGFEVQSLHPDKAQIPIVGSNGSSRLQDSRI
BetP      ----------------------------------------
```

**Fig A.** Multiple sequence alignment of dDAT, hNET, LeuT, GlyT2, GlyT1 and BetP.

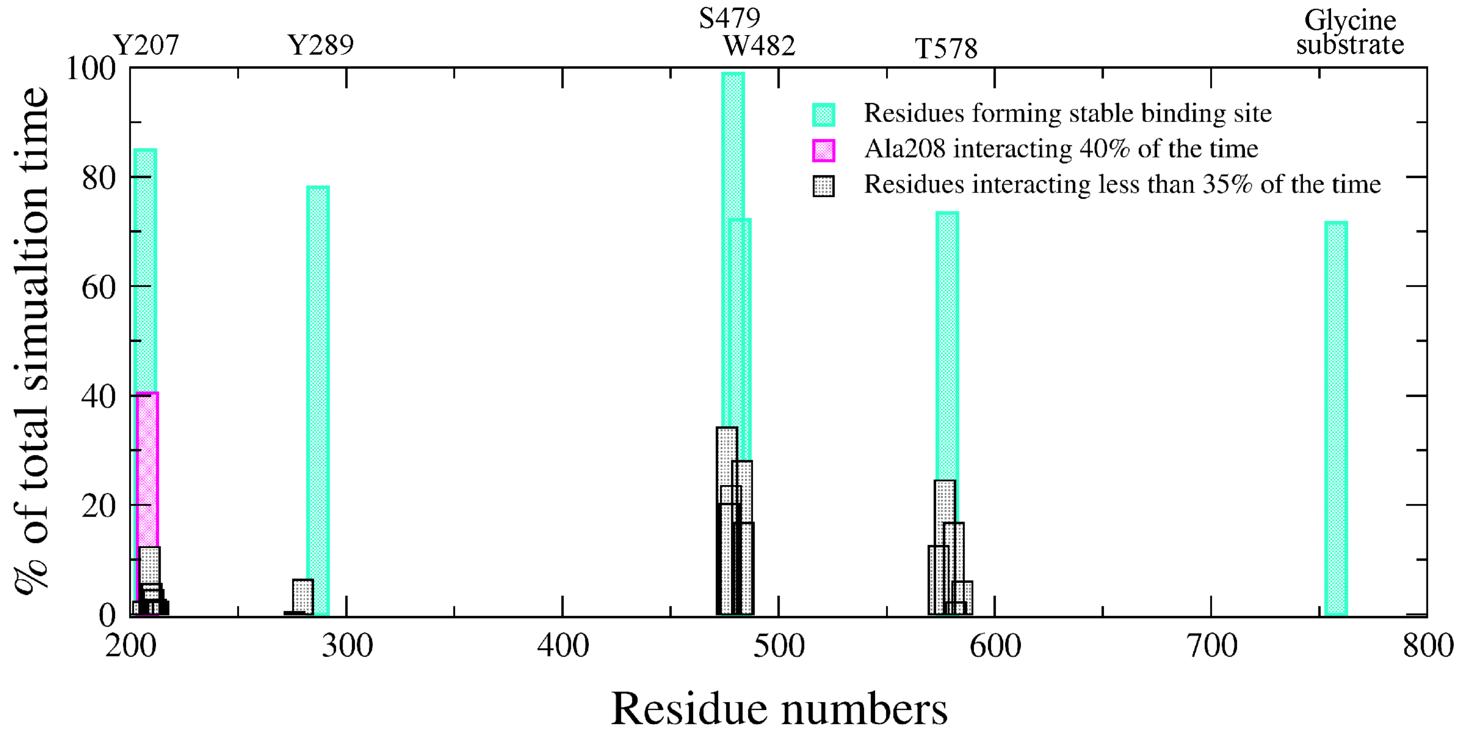


**Fig B.** GlyT2 residues forming direct contacts with the $Na^+$ ion residing at the Na1 site, averaged over the 250 ns of simulation time from the five 50 ns MD trajectories. A residue is considered to be forming a direct contact if its CA atom lies within a radius of 4.0 Å of the Na1 $Na^+$ ion. Note that the substrate glycine is included as the last residue in the protein.

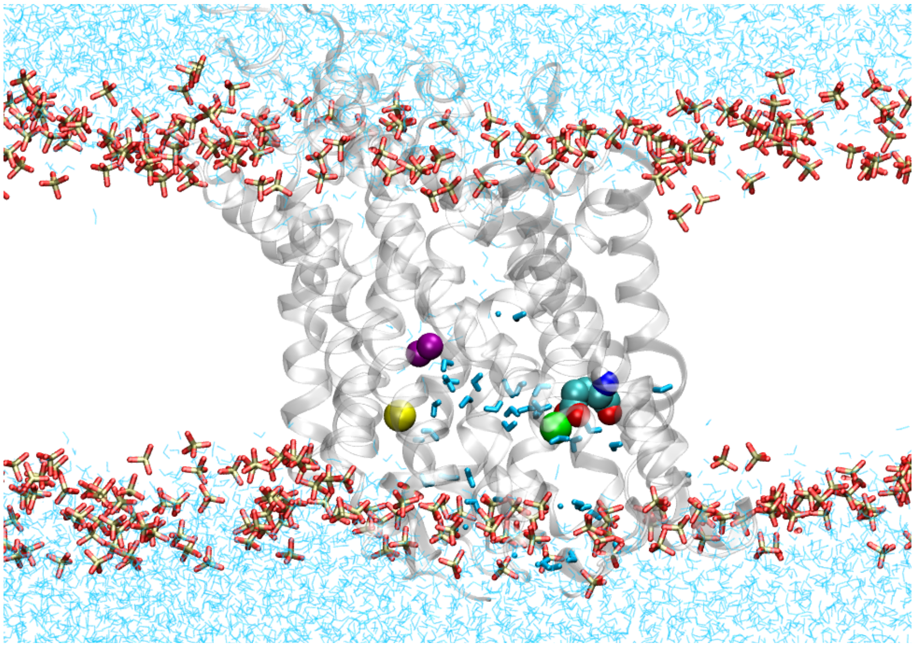

**Fig C.** A solvent-accessible cleft through the GlyT2 homology model allows the diffusion of $Na^+$ from its initial position (yellow spacefill) to its final position in MD simulations (green spacefill) corresponding to the location of proposed Na3 site. E648 and the lipid headgroups are shown as CPK licorice. Water molecules are colored cyan and those solvating the protein cleft are in licorice representation. The protein is shown as transparent ribbons.

**Table A.** Distance between the $Na^+$ ion occupying the Na1 site of GlyT2 and the binding site, residues averaged over all five 50 ns MD simulations.

| Residues | Distance (Å) |
|---|---|
| Y207 | 3.5 ± 0.6 |
| Y289 | 3.3 ± 0.6 |
| S479 | 2.8 ± 0.3 |
| W482 | 3.6 ± 0.5 |
| T578 | 3.9 ± 0.5 |
| Glycine substrate | 3.6 ± 0.4 |

**Table B.** Distance between the $Na^+$ ion occupying the Na2 site of GlyT2 and the binding site, averaged over all five 50 ns MD simulations.

| **Residues** | **Distance (Å)** |
| --- | --- |
| G206 | 2.6 ± 0.4 |
| V209 | 2.5 ± 0.3 |
| L574 | 2.8 ± 0.4 |
| D577 | 2.4 ± 0.3 |

**Table C.** Distance between the glycine substrate and the GlyT2 residues directly implicated in binding, averaged over all five 50 ns MD simulations.

| **Residues** | **Distance (Å)** |
| --- | --- |
| W215 | 3.3 ± 0.6 |
| Y286 | 3.6 ± 0.4 |
| Y287 | 2.6 ± 0.3 |
| G575 | 2.5 ± 0.3 |
| T578 | 2.2 ± 0.4 |

**Table D.** Distance between the $Na^+$ ion occupying the proposed Na3 binding site and the GlyT2 contacting residues, averaged over the last 35 ns of five MD simulations.

| **Residues** | **Distance (Å)** |
| --- | --- |
| E648 | 2.5 ± 0.3 |
| W263 | 3.2 ± 0.4 |
| M276 | 2.8 ± 0.4 |
| A481 | 2.9 ± 0.3 |
